# How do patients and physicians communicate about hereditary angioedema in the United States?

**DOI:** 10.1371/journal.pone.0260805

**Published:** 2021-12-02

**Authors:** Gagan Jain, Lauren Walter, Carolyn Reed, Patricia O’Donnell, Jeffrey Troy

**Affiliations:** 1 Takeda Pharmaceutical Company Limited, Lexington, Massachusetts, United States of America; 2 Verilogue, Philadelphia, Pennsylvania, United States of America; Mazzini Hospital, ITALY

## Abstract

**Background:**

Hereditary angioedema (HAE) is a rare disease that manifests as recurrent and debilitating angioedema attacks, significantly impacting patients’ quality of life.

**Objective:**

To assess communication dynamics between patients with HAE and treating physicians and the impact this has on the treatment of HAE in the United States.

**Methods:**

This observational study used an institutional review board–approved protocol to collect four sources of patient–physician communication data from the period between January 2015 and May 2017: in-office conversations between patients aged ≥18 years with HAE and physicians, follow-up dictations with physicians, telephone interviews with patients and physicians, and publicly available social media posts from patients. Participant language was qualitatively assessed and key communication elements and communication gaps identified.

**Results:**

Twenty-five in-office conversations, 14 follow-up physician dictations, and 17 telephone interviews were conducted with a total of 29 unique patients, 4 caregivers, and 14 physicians. In-office conversations were generally physician-driven and focused primarily on symptom frequency, location, and severity; lexicon from both parties centered on “episodes” and “swelling.” During visits, impact on quality of life was not routinely assessed by physicians nor discussed proactively by patients; however, during telephone interviews and online, patients frequently described the multifaceted burden of HAE. Patients highlighted the difficulties they experience by using repetition, emphasis, and metaphors; they also varied the descriptors used for attacks depending on the communication goal. Physicians used intensifiers to emphasize the necessity of rescue medication access, whereas prophylactic treatments were positioned as an option for frequent or laryngeal attacks.

**Conclusion:**

Vocabulary differences suggest that the full impact of HAE is not consistently communicated by patients to physicians during clinical visits, indicating the potential for misaligned understanding of disease burden. A patient-driven, rather than physician-driven approach to the discussions may elicit valuable information that could help to optimize treatment approaches.

## Introduction

Hereditary angioedema (HAE) due to C1 inhibitor deficiency or dysfunction is a rare disease characterized by recurrent swelling of subcutaneous or submucosal tissue [1]. HAE is caused by mutations in the *SERPING1* gene, with angioedema attacks primarily mediated by bradykinin; prevalence of HAE is reported to be approximately 1 in 50,000 [1]. HAE attacks are unpredictable in their frequency, location, and severity, and can cause disfigurement, disability, and pain [2]; furthermore, laryngeal attacks are potentially fatal [3].

The burden of disease for patients with HAE can be substantial, with significantly impaired quality of life (QoL), and higher levels of anxiety and depression compared with patients without HAE [4–6]. Impaired QoL and emotional wellbeing can continue even between attacks [4]. Previous research has led to the development of the Angioedema Quality of Life Questionnaire (AE-QoL), a tool to assess changes to angioedema patients’ QoL over time [7]. This tool, which covers a broad range of QoL components including mood, fears, and functioning, has been effective at measuring changes to QoL impairment over time, but the extent of its use among physicians treating HAE is unknown [8].

Patients have reported additional distress when physicians fail to provide an accurate diagnosis of HAE [6]; misdiagnoses were reported by almost half of 418 patients in one observational study, and delays in diagnosis of 13–20 years have been reported [9, 10]. There is also evidence of a considerable societal burden of HAE beyond affected patients [4, 11]. Absenteeism from work or school, reduced work productivity between attacks, and hindrance of professional and educational advancement have been reported among patients and caregivers [4, 11].

Available therapies for patients with HAE are categorized as on-demand treatment to be used in the event of an angioedema attack, and prophylactic treatment used to prevent or attenuate attacks [1]. Approved prophylactic treatments for HAE vary in their ability to prevent breakthrough attacks, and in their safety, accessibility, and cost [12–14]. The updated 2017 international World Allergy Organization and European Academy of Allergy and Clinical Immunology guidelines for the management of HAE recommend that patients are assessed for long-term prophylaxis at every visit, with disease burden and patient preferences being taken into consideration [1].

Effective communication between health care providers and patients is an essential component of successful care, particularly with respect to the lifelong management of a chronic illness [15–17]. Previously reported barriers to effective patient–physician communication include a perceived lack of time during office visits, use of “disease-oriented” rather than “patient-oriented” evidence when discussing treatment plans, and attitudes of health care providers [18]. Patients with chronic diseases experience unique challenges that can affect how they communicate with health care professionals, such as the psychological impact associated with diagnosis of lifelong illness, and geographical distance to a specialist physician [19]. Furthermore, patients with rare, chronic illnesses such as HAE may initially be in the care of non-specialist physicians who lack awareness of rare diseases, and they may feel the need to become more knowledgeable about their disease and potential therapies in order to obtain accurate diagnosis and management options. Effective exchange of information between patients and physicians can help physicians understand their patient’s preferences and offer options that optimally meet the patient’s needs.

Information on the interaction between patients with HAE and treating physicians is scarce; existing literature focuses on patients’ satisfaction with their treatment and/or experience with healthcare providers. A 2015 survey of 106 patients with HAE type 1/2 in the United States reported that 89.6% were satisfied with the care provided by their physician; 69.8% used prophylactic therapy and 88.7% used on-demand medication to treat attacks [20]. Only 39.6% of patients reported being satisfied with the care received in an urgent care center or emergency department. However, these data were derived from patients who attended the Hereditary Angioedema Association National Patient Summit where the survey was conducted, so results may not be generalizable to the overall population of patients with HAE. A 2013 survey of 245 physicians treating HAE in the United States reported that 40.3% felt that their patients were very satisfied with their treatment, and that only 2.7% thought that patients they had treated were not satisfied [21].

Patient-centered communication, where physicians examine both the disease and its impact on patients [22, 23], is increasingly encouraged. Key features of this communication style include open and nondirected conversation, and consideration of the patient’s psychological and social environment [22]. The impact of physician-centered versus patient-centered communication styles on the outcomes of medical encounters has been the subject of recent research [22]. However, despite efforts by physicians to “design” the encounters, the interactions remain inherently co-constructed by both patient and physician and “local negotiation” between doctor and patient have a marked effect on the direction of the dialogue compared to the physician’s previously established plan for the encounter [24]. Notably, focus on the patient’s role in co-constructing the in-office dialogue is limited in the literature [25] likely due to the scarcity of naturally occurring conversational data. Studies of communication dynamics in other chronic disease states have shown that trust and relational attachment with physicians are strongly associated with treatment adherence and, consequently, positive patient outcomes [26]; the same dynamic may also affect patients with HAE. Patient-caregiver-physician communication dynamics can determine the extent to which physicians are able to encourage caregivers and patients to explain their concerns, particularly with respect to QoL, and thus be able to improve disease management.

Herein describes an observational study that was conducted in the United States to understand communication patterns regarding HAE between and among patients with HAE, their caregivers, and physicians, in order to assess the impact of communication dynamics on disease management and to identify communication gaps so as to improve patient care. The study also offers a preliminary exploration of the situated and sequential nature of conversations between physicians and their HAE patients.

## Methods

This study was exploratory and informational in nature and employed the tenets of grounded theory, thus there were no objectively testable hypotheses associated with the following study aims:

Objective 1: Assess the communication dynamic between and among healthcare providers, patients, and caregivers (if present) when discussing HAE during office visits.
Objective 2: Assess the emotional impact of HAE on patients, as well as determine the impact of QoL discussions on disease management.
Objective 3: Identify relational and transactional communication dynamics that result in communication gaps or best practices to create solutions to improve patient care.
Objective 4: Assess how prescription treatment options are introduced.

### Study participants

Physicians were recruited for the study from a panel of community-based allergist practices who use the Verilogue Point-of-Practice database and technology system. The database comprised input from 1528 physicians in diverse specialties practicing throughout the United States [27]. Physicians were eligible if they reported treating patients with HAE at their practice. Geographically dispersed allergist practices were selected to account for potential regional differences. English-speaking patients aged ≥18 years with a diagnosis of HAE were eligible for the study. Patients were asked to participate in recordings upon presenting to their physician in-office. Patients in phone interviews were located in the United States and were recruited from Verilogue’s database of interactions. A sample size calculation was not performed for this study, as quantitative analyses were not conducted.

### Study design

Four data sources or communication settings were used: (1) in-office recordings of communication between participating physicians and patients with HAE (and caregivers if present) at a routinely scheduled clinical visit using a proprietary smartphone application—personal information was deidentified once recordings were transmitted securely along with the patient’s medical and demographic data; (2) follow-up physician dictations after the scheduled clinical visit; (3) follow-up in-depth telephone interviews with a subset of participating patients using preapproved interview guides, and with the same physicians from in-office recordings plus additional physicians who were treating patients with HAE at the time of the study; and (4) netnography or evaluation of online patient behaviors, utilized by selecting public social media posts (on Facebook, Instagram, Reddit, and Twitter) about the disease and related treatment from patients with HAE and advocacy groups associated with HAE. Data from in-office dialogues, follow-up physician dictations, and telephone interviews were collected between June 2016 and April 2017; netnography data posted between January 2015 and May 2017 were examined.

### Study approval and informed consent

The study protocol was approved by the Western Institutional Review Board. Written informed consent for study participation was secured before the recording of in-office dialogue and telephone interviews; verbal consent was also requested at the start of telephone interviews. Patients and/or their caregivers could request the termination of recordings at any time; if this occurred, the dialogue was not included in data analyses. All information from in-office recordings and telephone interviews was deidentified and anonymized before data analyses. For netnography, only publicly available data were analyzed, with no interaction between study investigators and the patients with HAE whose social media posts contributed to the study.

### Data analyses

Data from all four sources or settings were qualitatively assessed using a sociolinguistic framework. Audio recordings were transcribed and analyzed by trained linguists and analysts. Key communication elements such as frequently used words, phrases, topics, and conversational moves were identified. Conversation analysis was used to assess power dynamics and the way in which rhetorical style and information provision practices may affect the efficacy of communication. The ways in which physicians communicated various management and treatment recommendations were also investigated. Social media analysis was qualitative and ethnographic in nature; the number of unique posts or handles was not collected.

## Results

### Study participants by setting

In total, 14 physicians who were treating patients with HAE at the time of this study, 29 unique patients diagnosed with HAE, and 4 caregivers participated in the study. Patients who participated in in-office interviews (n = 24) ranged in age groups from 18‒24 years old to 75‒79 years old (Table 1). The age groups with the highest frequency were 30–34 years old (n = 6, 25.0%) and 40–44 years old (n = 5, 17.0%). Most patients were female (n = 17, 71.0%), White (n = 20, 83%), and had private medical insurance (n = 14; 58%).

**Table 1:** Demographics and characteristics of patients.

	N = 24
Age range, years, n (%)	
18‒24	2 (8)
25‒29	1 (4)
30‒34	6 (25)
35‒39	0
40‒44	4 (17)
45‒49	2 (8)
50‒54	1 (4)
55‒59	0
60‒64	3 (13)
65‒69	1 (4)
70‒74	3 (13)
75‒79	1 (4)
Sex, n (%)	
Female	17 (71)
Male	7 (29)
Ethnicity, n (%)	
White	20 (83)
Black	3 (13)
Latin-X	1 (4)
Insurance, n (%)	
Private (preferred provider organization, health maintenance organization)	14 (58)
Public (Medicare, Medicaid)	10 (42)

Twenty-five in-office dialogues were recorded with five physicians and 24 patients; one patient had more than one recorded communication, and four patients were accompanied by caregivers. The average duration of recorded dialogue was 18 minutes and 16 seconds, and no patients or caregivers requested the termination of a recording. Fourteen dictations by the same five physicians were recorded. Seventeen telephone interviews were conducted: seven with patients (two patients who had participated in in-office recordings and five additional patients), and ten with physicians (one physician who had participated in in-office recordings and nine additional physicians). All patients who participated in telephone interviews were female; no other demographic data are available for the five additional patients who participated in telephone interviews only.

### Communication by participant and setting

The communication setting did not influence the focus of HAE communication made by physicians, which was primarily on assessing symptom frequency, location, and whether attacks were severe enough to require treatment or lead to work absenteeism. Frequency and location of HAE attacks took precedence over severity. Physicians guided in-office dialogue by asking how often patients had attacks, how long they lasted, if patients were aware of any triggers, where attacks were located, whether patients had premonitions regarding impending attacks or prodromal symptoms, and whether attacks were treated. Physicians indicated during telephone interviews that the impact of HAE on patients’ QoL was not routinely discussed at office visits; furthermore, QoL burden was only assessed by physicians in terms of emergency department visits and work absenteeism in both clinical visits and telephone interviews.

For patients, the focus of their communication regarding HAE differed by communication setting. During in-office visits, patients focused on the nature of attacks and did not proactively share the impact of HAE on QoL. Patients were expected to drive the conversation and report symptoms and treatment history in order to evaluate treatment efficacy. Although patients did not proactively report on the impact on QoL during clinical visits, they indicated that they would be willing to discuss it if probed by the physician; however, dictations showed that physicians assumed patients were not burdened by HAE if it was not communicated without prompting. During telephone interviews, 5/7 patients reported that the burden of HAE was multidimensional and impacted their daily life in many ways. They also shared their anxiety about not having access to medication, even if symptoms were under control at the time. In social media posts, patients sought to raise awareness about HAE and highlight the multidimensional burden associated with HAE. Physical appearance during HAE attacks was highlighted as a challenge. Patients aspired to create a supportive community to share the challenges of the disease that they might otherwise face alone. Both patients and physicians highlighted the unpredictable nature of HAE attacks as the primary disease burden across all communications settings. Potentially fatal laryngeal attacks were most concerning for physicians and patients across all settings.

### Key communication themes

#### Rarity of HAE

Patients felt “isolated” because of the rare nature of HAE and found it difficult to communicate with family members and non-specialists because of others’ lack of understanding about the disease. Patients acquired the role of HAE expert and undertook the responsibility of educating others and “managing” their own care, sometimes leading to the use of unique lexicons to describe HAE. Patients also became accustomed to bearing their symptoms as “normal,” meaning they did not report all attacks or minimized severity when describing their attacks.

The process of HAE diagnosis was reported as “long” and “complex,” and especially difficult for patients without a family history of HAE. Interactions with non-specialist physicians were described as “fraught with misalignment on symptom assessment and diagnosis.” Physicians framed having a “correct diagnosis” as a huge relief for patients; however, patients at no point suggested that diagnosis was a relief.

#### Unpredictability of HAE attacks

The greatest source of worry for both patients and physicians was identified as the unpredictability of attacks and their potentially life-threatening nature. To describe this unpredictability, patients used terminology such as “never know when,” “out of the blue,” “nobody knows,” “just shows up,” “can’t predict that,” and “comes and goes.” Physicians described this unpredictability in dictations using terminology such as “no specific triggers” and “can’t explain the increased frequency of attacks.” Among the burdens of HAE, patients included frequent last-minute cancellation of social plans, absence from employment or school because of symptoms and/or time spent in the emergency department, and expressed a fear of unexpected attacks occurring while traveling. Furthermore, when describing their social interactions, patients reported experiencing “guilt” whilst having difficulty in discussing an unexpected event or symptom, and consistently reported that symptoms negatively impact dynamics at social events or dictate their conversation topics.

#### Treatment of HAE

Patients expressed feeling “tethered” to their medication and “reliant” on others for administration of treatments; such treatments were reported by 5/7 patients as “inconvenient” and “expensive.” Patients reported that hospitals do not always stock HAE treatments and expressed “fear” and “frustration” owing to inconsistent emergency care by non-HAE specialists. Patients reported not treating all HAE attacks because they were unwilling to “waste” rescue medications and preferring to use them only if an attack was “bad enough.” Patients waited for “multiple” signs and symptoms or an obvious serious impact such as laryngeal swelling, and/or sought confirmation of attack severity before using rescue medication. Patients with symptoms that were perceived to be “controlled” and those who had safety concerns about switching treatments showed a reluctance to try new medications. None of the patients reported being told about the efficacy duration of prophylactic treatments.

Physicians reported advocating for a protocol for use of rescue medications, but inconsistent use of such protocols by patients was reported, and not all patients reported having a formal treatment protocol. Physicians indicated that a detailed discussion about treatment options was necessary because patients did not always “know much about treatments.” According to physicians, the treatment options were presented neutrally and informationally, without added evaluation or physicians’ preference. Rescue medications were positioned as a “need,” and physicians used intensifiers to present strong recommendations for acute treatment (Table 2). Prophylactic treatment was positioned as “optional” and based on frequent episodes, laryngeal attacks, or patient preference. The distinction between “short-term” and “long-term” prophylactic treatment was not discussed with patients during clinical visits, but physicians indicated during telephone interviews that it was discussed. Discussion of long-term prophylaxis was most likely to occur if patients communicated that HAE episodes were frequent, or if swelling occurred in the throat or on the lips. Physicians differentiated between mechanisms of action of treatment and route of administration but not between brands.

**Table 2:** Lexicon used by physicians to describe and recommend HAE treatments.

Treatment Topic	Language Used	Examples of vocabulary used
Rescue treatments	Physicians used intensifiers to emphasize the importance of having access to rescue medications. This was perceived as a strong recommendation	“Strongly advise”
		“I feel nervous”
	Physicians reported advocating for a protocol for use of rescue medications	“As needed”
		“If/in case you have an episode”
		“Emergency”
		“Acute”
		“On demand”
Prophylactic treatments	Physicians presented prophylactic treatments in a passive way, which may be perceived as a weak recommendation	“An available option”
		“Do you think”
		“Given a choice”
	Physicians described the purpose of prophylactic treatment as “prevention,” but the distinction between “short-term” or “long-term” prophylactic treatment was not discussed	“Prevention”
		“Preventive”
		“Preventative”
		“Prophylaxis”
		“Short- or long-term preventive treatments”
Mechanism of Action	Physicians differentiated between mechanisms of action of treatment but not between brands	“Replace the enzyme that your body is missing”
		“Block the enzyme that is involved in producing angioedema”
		“A receptor blocker”
Route of administration	Physicians differentiated treatments based on their route of administration	“IVs”
		“Subcutaneous”
		“Easiest”

HAE, hereditary angioedema; IV, intravenous.

### Vocabulary used to describe HAE

The vocabulary used to describe HAE symptoms by both patients and physicians during in-office dialogue was similar, with a focus largely on the “episodes” of “swelling.” During clinical visits, patients discussed “tight” and “tingling” feelings; however, in social media posts, “pain” was more frequently mentioned. Physicians queried attack frequency by asking “how often” they occurred and “how long” they lasted, asked about attack location with a focus on tongue or throat swelling, and inquired about attack severity via asking whether there was a need for hospitalization or acute treatment.

Analysis of patients’ descriptions of their HAE symptoms revealed both intensifying discourse strategies as well as minimizing ones (Table 3). Patients used repetition, metaphors, and emphasis to evoke more powerful descriptions and convey the true burden of their condition. However, they also used minimizers when reporting their symptoms to downplay the impact of their symptoms.

**Table 3:** Lexicon used by patients to describe HAE.

Discourse Strategy	Lexicon class	Description	Examples of vocabulary used
Intensifying	Repetition	Patients used repetition of the same words to emphasize a point	“Doctor after doctor after doctor…”
	Metaphors	Patients used metaphors to visualize and create a powerful description of their symptoms and feelings	“Blew up like a balloon!”
			“Football”
			“Floater”^a^
			“From a different planet”
	Interjections	Patients used interjections to counter a default assumption of mild symptoms	“*Oh my gosh*, it was every other day”
	Tone	Patients altered their tone of voice to emphasize the intensity of their experience	“It goes ber-*serk*”
			“This *whole* side”
Minimizing	Framing symptoms comparatively	Patients compared their most recent symptoms to a real or potential worsened state	“Just once in a while”
			“Not as bad as it used to be”
	Down toners and negation	Patients negated the frequency and severity of their symptoms when these symptoms fell within their “new normal”	“A little”
			“Not extreme”
			“That’s all…”
			“Not every day”
			“Just discomfort”
			“Not very often”
	Focusing on swelling symptoms as short-lived	Sharing that symptoms resolved quickly minimized the impact that even short-lived symptoms could have on patients	“Didn’t last long”
			“It was gone away…”

^a^Spoken by a patient who was describing a family member’s description of them.

### Observations of communication gaps and opportunities

Seven gaps were identified where inconsistencies or barriers to effective communication between patients and physicians were observed (Table 4). Opportunities to address each of these communication gaps were proposed and include an agreed-upon lexicon, alignment of the focus of HAE communication, a mechanism to capture potential triggers of attacks, and a mutually agreed plan on how to manage attacks. Communication barriers to treatment may need to be addressed in order to ensure that patients receive optimal benefits from available therapy options.

**Table 4:** Observations of communication gaps and opportunities to improve patient–physician communication regarding HAE.

Observation category	Communication gaps and opportunities
Inconsistent choice of words by patients to describe attacks	An agreed-upon lexicon may be helpful for the physician to understand and appreciate the burden across different patients
Variation in focus of communication by setting	Alignment on the focus of communications between patient and physician may drive productive dialogues
Use of minimizers to downplay attacks or not always reporting symptoms	Reframing of the frequency and severity of attacks by the physician during probing (e.g. every other day reframed as “half the time”). Caregivers could also supplement the information by providing an “eyewitness account”
Unknown triggers	A mechanism to capture triggers or situations that preceded an attack could increase awareness and may aid in-office conversations
Communication barriers to treatment	Patients expressed a reduced willingness to try newer or improved therapies owing to at least one of the following reasons:
	1. Perceived control with current therapy (e.g. “got something that’s already working,” “can’t risk”)
	2. Difficulty in treatment administration (e.g. “can’t stab/inject myself,” inconvenience of frequent administration of prophylactic treatments)
	3. Logistics and access to treatment (e.g. need for travel to receive therapy, challenges in getting approval by payers, access to limited quantity of acute therapy)
	4. Concern over long-term safety of some treatments
Interpersonal communication difficulties	Though HAE affects day-to-day activities, one of the most burdensome issues is talking about the condition with loved ones. Patients expressed feelings of “guilt” that their HAE can negatively impact the dynamics at social events. Attempts to address these difficulties included:
	1. Online “swell families” that were described as educational and served as a positive interpersonal resource and support network for patients
	2. The taking of “swelfies” to highlight natural beauty and/or appreciate healthy days. Physical appearance owing to symptoms can be an initial challenge for patients’ social relationships, but according to the study findings, is also becoming a point of pride in online posts. This type of patient-driven social media appears to be causing a shift in perception among patients
	3. Patients with HAE (online identities) highlighting what non-HAE activities they are passionate about (self, first), followed by raising awareness for HAE (disease, second)
Lack of a plan	Having an agreed-upon plan to manage HAE attacks and communicating the plan with family members and providers (e.g. emergency or primary care physicians) may limit miscommunication

HAE, hereditary angioedema.

## Discussion

This qualitative analysis of dialogue during clinical visits, follow-up telephone interviews, and patient netnography highlighted discrepancies in the way in which HAE is communicated by diagnosed patients and treating allergists. Three key communication themes were identified, with dialogue focused on the rarity of HAE, the unpredictability of attacks, and the treatment of HAE. Key patient burdens included feelings of isolation, fear of attacks, responsibility for disease education, frustration over inconsistent emergency care, and guilt regarding limitations due to HAE. Four caregivers were present during office visits and were able to provide eyewitness accounts to highlight disease burden, sometimes being the first to mention the patient’s symptoms. To the best of our knowledge, this is the first study to assess communication between patients with HAE and treating physicians.

During office visits, patients and physicians were misaligned with regard to how they discussed HAE and disease burden. Physicians focused on symptom severity and frequency, and although patients were willing to share the multifaceted nature of disease burden during telephone interviews and on social media, vocabulary concerning QoL was less readily used during direct communication with physicians. Although physicians recognized that uncertainty was a great burden for patients with HAE, and patients expect physicians to ask about their concerns outside of those relating directly to disease symptoms [22], addressing the sustained functional and emotional impact of uncertainty was not the focus of in-office dialogue or a goal of treatment. Patients may also underreport symptoms during clinical visits because they have become accustomed to the burden, and only describe experiences that deviate from their “new normal.” To address this, physicians could challenge patients to report the accurate impact of HAE by reframing minimized communication, e.g. a patient’s use of “just some nights” could be reframed by the physician’s reply of “three times a week is about 50% of the time.” A patient’s perception of disease etiology is affected by the choice of words to describe the disease and treatment, which in turn affects a patient’s intention to undergo treatment [28]. This issue is complex for rare diseases such as HAE where healthcare providers have limited interaction opportunities and disease-specific communication strategies. Understanding of the etiology of HAE and treatment options are evolving continuously, and if the description of HAE-related symptoms and patient experiences are communicated and understood similarly by the physician and patient, improvement in disease outcomes and patient satisfaction may be observed [29].

The findings from this study point to doctor-centered communication practices [22] when dealing with patients with HAE, as treatment options were offered on the basis of symptoms and efficacy of prior therapy, even though a patient-centered approach (whereby patients’ needs beyond symptomology are openly discussed and considered in the treatment plan) would be more suitable given the complexities of living with HAE. Physicians indicated during telephone interviews that they expected patients to proactively report the impact of HAE on their QoL; however, in-office dialogue shows that patients rarely volunteered this information. Probing of patients’ QoL and functional or emotional status during and between attacks, or widespread use of the AE-QoL questionnaire [7, 8], could improve physicians’ understanding of the perpetual nature of the burden of HAE.

The onset of HAE symptoms establishes a journey for patients that is experienced by many with orphan diseases, whereby self-evaluation of health status initiates the seeking of medical advice; patients may proactively search for information on the internet and through social networks [30]. In this study, netnography showed that patients with HAE sought to increase awareness of the disease and its burden through their online activity. Social media posts may also help patients communicate more effectively with family and friends, and alleviate feelings of guilt experienced when symptoms impact social events, work, or education. Patients also felt that they were responsible for educating nonspecialist health care providers. Increased efforts to raise awareness of HAE among emergency department physicians and in other areas of health care may reduce some of this burden [31].

A significant body of work has contributed to understanding how patient narratives and stories function in the context of the medical encounter. Most relevant to this study, Ainsworth-Vaughn [32] showed that physicians and patients use stories to “propose, argue against, augment, or accept (i.e., to co-construct) an overarching diagnostic hypothesis and its associated treatment plan.” Stories have also been shown to function as a way to assert a version of oneself that had previously been suppressed within the institutionalized structure of the discourse [33, 34], however other research demonstrates that patients generally have limited success in securing the physician’s respect for these other “life worlds” [35, 36].

When the balance of power between a patient and physician is considered, along with the ways in which that power manifests in dialogue, space in the discourse for such stories may be limited. Not only do patients and physicians have unequal rights to ask questions, but physicians also have more power when it comes to initiating and concluding the interaction [32]; multiple studies have shown that medical encounters often consist primarily of physicians asking questions and patients answering them [37, 38]. Further, the questions that interlocutors (including physicians) ask, even those that are open-ended, necessarily restrict the topic of their conversational partner’s (i.e., the patient’s) response [39]. It is this control over the talk that may impact the subsequent outcomes of the interaction. For example, Kaplan et al. [40] showed that certain types of talk within a medical encounter were associated with improved chronic health, including improved blood pressure or blood sugar readings. We found that in-office dialogues were more physician-driven, which often constrained patients’ abilities to share information in a narrative format, thus hindering a rich resource for informing treatment discussions. However patients’ stories, which surfaced elements of their “life world” that highlight the impact of HAE on their QoL, were more prominent in telephone interviews and on social media.

The communication dynamics observed herein may impede the effective exchange of information between parties. For example, the time-limited nature of these interactions may have led physicians to use a variety of linguistic strategies to constrain the number and duration of conversational turns a patient/caregiver takes, as too many or very long turns could have slowed the encounter, impeding the exchange of very important information providers need in order to make sound treatment decisions. Indeed, the need for additional time was mentioned as a barrier to shared decision making in HAE [41]. These linguistic practices can also lead physicians and patients/caregivers alike to self-edit, restricting exchange of relevant information and negatively affecting patients’/caregivers’ question-asking behavior [42, 43]. These restrictions can lead to misinterpretation, minimization of patient concerns, general lack of patient engagement in decision-making, uncertainty about expectations, and dissatisfaction with care, among other negative outcomes.

More effective and patient-centered communication between patients and physicians has been shown to improve symptom relief, patient satisfaction, and adherence to treatment [44–46]. For rare diseases such as HAE, this is of even greater importance because of additional challenges faced by patients, such as the time taken to obtain an accurate diagnosis. Communication that focuses on tongue or throat swelling while minimizing other swelling and the functional and emotional burden of HAE may result in patients missing out on the benefits of new and existing treatment options. Patient attitudes towards treatment have been shown to be positively impacted by physicians who adjust their communication styles based on patients’ need salience [47]. In addition, Bientzle et al. [48] showed that although physicians generally respond to patient queries in a scientific, evidence-based manner, they tend to use emotional terms in response to emotional queries. Thus, physicians treating HAE need to be perceptive of their patients’ needs and flexible in their manner of communication. Although broad communication-skills training for physicians has been reported to improve patient satisfaction [49], the recommendations generated through this study could further advance disease management and improve patient–physician interaction specifically with regard to HAE. The creation of comprehensive and tailored treatment plans for HAE have previously been suggested [50, 51]; however, this study shows that the “lack of a plan” remains a communication gap that should be addressed to optimize disease management for patients with HAE. Literature for other chronic, QoL-impacting illnesses such as hemophilia, for example, tends to focus on general communication issues such as patient information preferences [43, 52], methods for delivering unpleasant news [53, 54], and the content of educational messages [55]. Furthermore, the nature of communication surrounding supportive HAE therapies and QoL issues, as well as ways to support physicians in discussion around HAE-related QoL issues, remains underexplored. The findings from this study provide important insight into the margins of HAE care, and enable a more productive dialogue around QoL issues.

The limitations of this study include a small sample size that is qualitative in nature. As part of the study methodology, participating physicians self-selected which of their treated patients to include. From the recordings, dialogue that best addressed the research objectives were selected for analysis. Future studies could address these limitations by including a random selection of dialogues and a greater sample size of participating physicians. The study was conducted before the availability of subcutaneously administered long-term prophylactic treatments [1, 56]; this, in addition to potential access issues, have contributed to a changing therapeutic landscape in recent years that may influence the outcomes of future studies of this nature in patients with HAE.

Analysis of patient–physician dialogue in this study demonstrated a need for more effective communication on the multifaceted burden of HAE to improve patient assessment and better inform treatment choices. Communication must be approached in a balanced manner, from both a scientific perspective as well as an emotional one. Physicians are guided by professional bounds and thus strive for accuracy during communications with patients, and must minimize their personal attitude towards health-related problems. On the other hand, patients are more emotional in the expression of their disease, which may or may not be an accurate reflection of their situation. This results in a communication gap that can hinder ideal disease management outcomes. Further exploration of the function of HAE patients’ narratives in the office and the relative utility of various strategies used by physicians to elicit, and thus have access to, patient stories would be valuable. Alignment on the vocabulary used to describe symptoms and discussion of the effect of HAE beyond angioedema attacks on patients’ daily life could help to optimize disease management and alleviate patient burden.
